# Medication Persistence Rates and Factors Associated with Persistence in Patients Following Stroke: A Cohort Study

**DOI:** 10.1186/1471-2377-8-25

**Published:** 2008-07-10

**Authors:** Heather L Lummis, Ingrid S Sketris, Gordon J Gubitz, Michel R Joffres, Gordon J Flowerdew

**Affiliations:** 1 Pharmacy Department, Capital District Health Authority, Halifax, Canada; 2 College of Pharmacy, Dalhousie University, Halifax, Canada; 3 Department of Medicine, Dalhousie University, Halifax, Canada; 4 Faculty of Health Sciences, Simon Fraser University, Burnaby, Canada; 5 Department of Community Health and Epidemiology, Dalhousie University, Halifax, Canada

## Abstract

**Background:**

Medication nonadherence can be as high as 50% and results in suboptimal patient outcomes. Stroke patients in particular can benefit from pharmacotherapy for thrombosis, hypertension, and dyslipidemia but are at high risk for medication nonpersistence.

**Methods:**

Patients who were admitted to the Queen Elizabeth II Health Sciences Centre in Halifax, Nova Scotia, with stroke between January 1, 2001 and December 31, 2002 were analyzed. Data collected were pre-stroke function, stroke subtype, stroke severity, patient outcomes, and medication use at discharge, and six and 12 months post discharge. Medication persistence at six and 12 months and the factors associated with nonpersistence at six months were examined using multivariable stepwise logistic regression.

**Results:**

At discharge, 420 patients (mean age 68.2 years, 55.7% male) were prescribed an average of 6.4 medications and mean prescription drug cost was $167 monthly. Antihypertensive (91%) and antithrombotic (96%) drug use at discharge were frequent, antilipidemic (73%) and antihyperglycemic (25%) drug use were less common. Self-reported persistence at six and 12 months after stroke was high (> 90%) for all categories.

In the multivariable model of medication nonpersistence at six months, people aged 65 to 79 years were less likely to be nonpersistent with antihypertensive medications than people aged 80 years or more (Odds ratio (OR) 0.11, 95% Confidence Interval (CI) 0.03–0.39). Monthly drug costs of < $90 or $90–199.99 were associated with greater nonpersistence, compared to monthly drug costs ≥$200 (OR 6.74, 95% CI 1.32–34.46 for < $90; OR 5.25, 95% CI 1.14–24.25 for $90–199.99). For the antithrombotic drug category, people aged 65 to 79 years were less likely to be nonpersistent than people aged 80 years or more (OR 0.23, 95% CI 0.06–0.81), and people who were disabled before admission were more likely to be nonpersistent than those not disabled (OR 7.01, 95% CI 1.66–29.58).

**Conclusion:**

Patients reported high medication persistence rates six and 12 months after stroke. Identification of factors associated with nonpersistence (such as older age and prior disability) will help predict which patients are at higher risk for discontinuing their medications.

## Background

Stroke is the third leading cause of death worldwide, responsible for 10% of all deaths.[1] Every year, 15 million people will have a stroke, of whom five million will die, and another five million will be permanently disabled. In North America, just over 15,000 people in Canada and 160,000 in the United States, died in 2000 from a stroke. Total costs for stroke, such as medical care and lost productivity, were estimated to be US $53.6 billion in 2000 for the United States.[1]

Persons recovering from a stroke or transient ischemic attack (TIA) are at high risk for recurrent stroke, disability, institutionalization, and death.[2] Pharmacotherapy that targets hypertension, vascular disease, and hyperlipidemia can decrease the risk of further vascular events and mortality.[2] Risk factor management in stroke patients has been the subject of numerous randomized clinical trials and meta-analyses of these trials. [2-13] These studies have shown that for people with atrial fibrillation and previous TIA, anticoagulant use can reduce recurrent stroke by two-thirds, and all vascular events can be reduced by one-half.[6] For patients with stroke in normal sinus rhythm, antiplatelet agents (such as acetylsalicylic acid, ASA) decrease the relative risk of stroke by 24%.[7] A 22% overall odds reduction of serious vascular events (non-fatal myocardial infarction [MI], non-fatal stroke, or vascular death) has also been attributed to antiplatelet therapy.[8] Blood pressure (BP) reduction for secondary prevention can reduce the relative risk of stroke by 22% for a 10 mmHg systolic BP reduction.[9] Results are similar regardless of the type of medication used.[9] Statins (a family of lipid-lowering agents such as atorvastatin and simvastatin) have been shown to produce an approximate 25% relative risk reduction of stroke in patients with a history of stroke or TIA.[10,11]

Despite the proven efficacy, medication nonadherence for patients with chronic diseases can be as high as 50% and is the main reason why patients do not achieve maximum clinical benefit.[14] Medication persistence, or continuing to take a medication long-term, is one aspect of medication adherence. Stroke patients are potentially at high risk for medication nonpersistence because they require long-term drug therapy, are more likely to have cognitive or physical impairments, and are often depressed.[15] A recent study investigated persistence of lipid lowering therapies after stroke and found that 39% of patients had discontinued therapy at one year after discharge.[16]. Medication persistence rates after stroke have varied from 37–96%, depending on the medication, in the small number of studies that have examined this issue. [16-21] One study that analyzed patient variables associated with medication use found that older age and a cardioembolic cause of stroke were significantly predictive of higher medication compliance.[19]

It is unclear how well patients persist in taking their medications at one year after discharge from a Canadian acute stroke unit. As well, understanding potential factors that may affect persistence can assist health providers in supporting those patients at risk of stopping their medications. Our objectives were to report medication persistence rates for four important drug categories at six and 12 months after stroke, and analyze the potential demographic and clinical factors associated with medication persistence at six months.

## Methods

The study population were stroke patients prospectively enrolled in the Stroke Outcome Study (SOS) at the Acute Stroke Unit (ASU) of the Queen Elizabeth II Health Sciences Centre (QEII HSC) between January 1, 2001 and December 31, 2002 and followed for one year. The QEII HSC is a 1000 bed, tertiary care and referral centre for the Canadian Atlantic provinces. The ASU treats 25% of all patients who have a stroke in Nova Scotia. The acute stroke team includes neurologists, nurses, dieticians, and physiotherapists who are trained in acute stroke management. A pharmacist provides pharmaceutical care as part of the healthcare team and conducts follow-up visits in the stroke clinic which focus on medication management and compliance. Medical treatment is standardized by using preprinted order sets and preprinted interim discharge reports for family physicians. A description of the care received and patient outcomes has been published.[22]

The SOS data collection occurred during hospitalization, six months, and 12 months after stroke. Data collected during the hospitalization included demographics (age, sex, marital status, postal code), preadmission disability (Oxford Handicap Score [OHS][23], previous history of stroke, mortality, stroke recurrence, length of stay, stroke subtype (Oxford Community Stroke Project [OCSP] subtypes[24]), stroke severity (First Stroke Severity Score [FSSS][25]), functional status (Oxford Handicap Score and Barthel Index[26]), cognitive status (clock drawing[26] and orientation to person, place, time[27]), discharge medication list and discharge disposition (discharged home, to a long term care facility, etc.). Age was categorized into < 65 years old, 65–79 years old, and 80 years and greater. The OHS functional disability score was categorized into dependent on others for activities of daily living (score 4–5) or independent (score 0–3). OCSP stroke subtypes for ischemic stroke were defined as lacunar stroke (LACS), partial anterior circulation stroke (PACS), posterior circulation stroke (POCS), total anterior circulation stroke (TACS), transient ischemic attack (TIA) or uncertain. Stroke severity was defined as a FSSS score of mild (score 1–4), moderate (score 5–7), severe (score 8–10), or TIA (score 11). Cognitive impairment, measured by the clock drawing test during the patient's hospitalization, was also converted into a dichotomous variable where a score of ≤ 12 meant impairment, and scores > 12 meant normal cognitive functioning. Medication use before admission to the hospital was not recorded.

Data collected at six months were mortality, stroke recurrence, disposition, functional status (modified Rankin scale [MRS][28] and Barthel Index), and self-reported medication use. The MRS functional disability score was categorized into dependent on others for activities of daily living (score 4–5) or independent (score 0–3). Medication use was collected by the study coordinator who made all attempts to be as complete as possible by asking for help from caregivers, asking the names to be read from medication bottles or brought in to the study visit, obtaining medication lists from nursing homes, and calling pharmacies for verification if there was any doubt in the accuracy of the list. Data collected at 12 months were mortality, stroke recurrence, disposition, functional status (MRS and Barthel Index), cognitive status (clock drawing and orientation to person, place, time), depression score (Geriatric Depression Scale[29]), dementia score (Global Deterioration Scale[30]), and self-reported medication use. However, not all variables were needed for the medication persistence sub-study reported here.

The SOS Study did not collect any clinical information such as blood pressure measurements, lipid profile tests, health resource use, or lifestyle modifications after the stroke which could include exercise, smoking cessation, or diet changes. Medication use was recorded but reasons why the drug regimen changed, such as due to adverse effects, were not obtained from patients or their family practitioners. As well, how much patients paid for their medications out of pocket was not obtainable for this study.

Inclusion criteria for the medication persistence sub-study were:

• Diagnosed ischemic stroke – stroke type was determined by the attending neurologist after thorough neurological exam and CT scan to rule out hemorrhagic stroke or tumor,

• Survived the hospitalization,

• Medication list available at discharge and six months, and

• Completed the 12 month visit or had died by 12 months.

Ethics approval was received from the Capital Health Research Ethics Board on January 13, 2005 and the study was conducted according to institutional guidelines.

### Additional variables

Variables needed for the medication persistence sub-study were then added to the SOS dataset. Medication use had been recorded on paper so medication names, dose and directions at discharge, six months, and 12 months were then entered into a Microsoft Excel spreadsheet (Version 11.0, Microsoft^® ^Office Excel 2003, Redmond, Washington, US). Medications were coded according to the 2004 World Health Organization's (WHO) Anatomical Therapeutic Chemical (ATC) System, which categorizes pharmaceuticals according to the organ or system on which they exert their effect and their specific chemical properties.[31] All antihyperglycemic (group A10), antithrombotic (B01), antihypertensive (C02 – C09) and antilipidemic (C10) medications were coded at discharge, six months, and 12 months for each patient.[31]

Monthly drug costs for prescription drugs, excluding pharmacists' professional fees, were estimated from the patient's discharge medication list. Prices were obtained from the January 2005 Atlantic Pharmaceutical Services Incorporated (APSI) Pricing Guide[32] (2617 drugs or 99.3%), from PPS Pharma 2002[33] (two drugs), PPS Pharma 2000[34] (three drugs) and the hospital pharmacy price (13 drugs). Costs were conservatively estimated by using the lowest price per unit (brand or if off-patent the lowest generic price). Missing directions were obtained from the hospital computer system (78 drugs) or by using the WHO Defined Daily Dose (DDD)[31] (20 drugs). The WHO defines the DDD as "the assumed average maintenance dose per day for a drug used for its main indication in adults".[31] If a reported dose appeared to be an outlier (greater than twice the upper limit or less than half of the lower limit of Health Canada's approved dose), the WHO DDD was used to estimate the cost (one drug). Antibiotics were costed for five days duration only. Drug costs were then categorized into three groups: < $90/month, $91–199.99/month, or $200 or greater/month.

Patients were considered to have diabetes or atrial fibrillation if the diagnosis was coded from the hospital's health records data as a co-morbidity or secondary diagnosis during the hospitalization for stroke. The International Classification of Diseases diagnosis codes for diabetes were 250 (9^th ^version) and E10, E11, or E14 (10^th ^version). The codes for atrial fibrillation were 427.3 (9^th ^version) and I48 (10^th ^version).[35] Diabetes could be treated with pharmacological agents or diet controlled. The actual date of diagnosis for the comorbidity was not available. Tobacco use at the time of admission was obtained from the hospital discharge report. The Population Health Research Unit at Dalhousie University, Halifax, Nova Scotia[36] provided average household income by linking the patients' postal codes to 2001 Canadian Census data. Average household income was unavailable for 22 patients (5.4%) residing in a geographic area with a population of less than 250. The number of non-research related visits to the QEII HSC Neurology clinic between the patient's discharge and 12 month visit were documented from the hospital pharmacy computer system.

### Study outcome

The outcome measure was medication persistence for the antihypertensive, antithrombotic, antilipidemic, and antihyperglycemic drug categories. Patients were classified as persistent or nonpersistent at six months by comparing the discharge medication list with the six month medication list. Patients were classified as persistent or nonpersistent at 12 months by comparing the discharge medication list with the 12 month medication list, if it was available. Since patients derive benefit if they continue taking any drug within the drug category of interest (eg. antihypertensive), we considered patients to be persistent for the drug category, not for the individual drugs. Patients who switched from one drug to another were considered persistent for the purposes of this study. This method will provide high estimates of persistence but will reflect a better "real-world" analysis of whether patients continue to take their medication after stroke.

### Statistical methods

The multidimensional WHO model of factors that affect medication adherence were utilized as a guide to analyze medication persistence.[14] Logistic regression was used to test variables for univariate associations with medication persistence. The following variables were tested in each drug category: age, sex, marital status, household income, tobacco use, number of medications or doses taken daily, medication costs, previous stroke history, atrial fibrillation, diabetes, stroke subtype, stroke severity, functional disability, and cognitive status during hospitalization. Variables associated at the 10% level of significance were entered in a multivariable stepwise logistic regression model. Effect modifiers were identified using the Breslow-Day test for homogeneity of the odds ratio, with p < 0.05 indicating the existence of effect modification. If this occurred, the regression model was stratified by the effect modifier. All analyses were performed using SAS software (version 8.02, SAS^® ^Institute, Cary, North Carolina, US).

## Results

### Demographics

**Figure 1:**
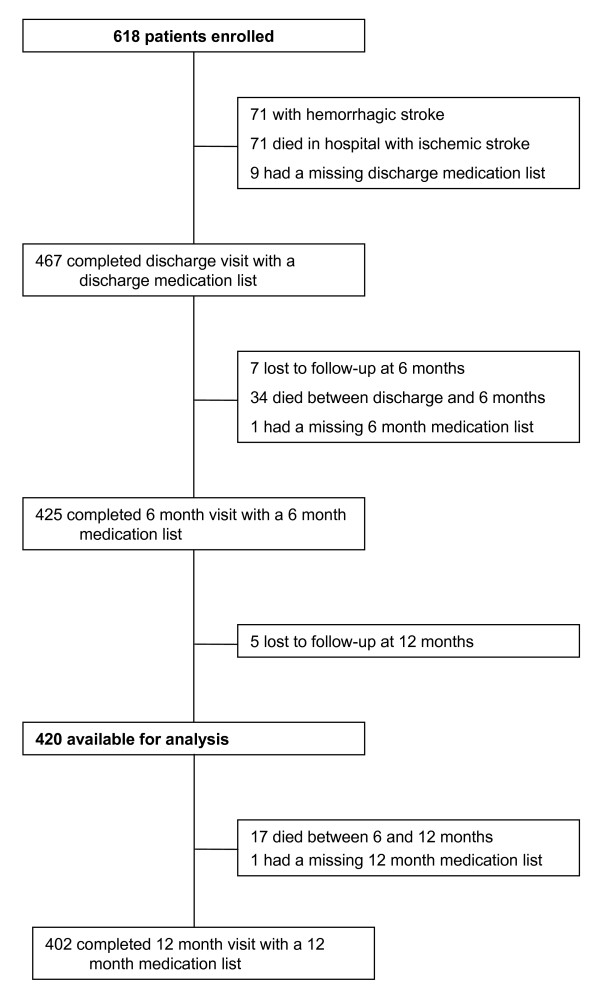
Study Flow Diagram of the 420 Eligible Patients in the Medication Persistence Analysis of the Stroke Outcome Study at the Queen Elizabeth II Health Sciences Centre, Halifax, Nova Scotia, Canada.

There were 671 patients admitted with stroke to the ASU between January 1, 2001 and December 31, 2002, of whom 618 (92%) gave informed consent for the study. Of these, 198 patients were excluded due to hemorrhagic stroke (71), death between hospital admission and the six month visit (105), missing medication list (10) and loss to follow-up (12) (Figure 1). This left 420 patients available for analysis for medication persistence at the six month visit (Table 1). At 12 months, a further 18 patients were not eligible (17 had died and 1 was missing a medication list), leaving 402 patients available at the last visit. The mean age was 68.2 years (standard deviation 13.8) and 234 (55.7%) were male. There were 145 patients (34.5%) who returned for follow-up to the neurology clinic in the first year after discharge. These follow-up visits were part of standard care and not required by the research study. Half of the patients did not have cognitive testing performed (53.6%) either because the patient was not able to perform the test (eg. very severe illness) or the patient was discharged before the test could be administered (eg. very mild illness).

**Table 1:** Characteristics of the 420 Patients Enrolled in the Stroke Outcome Study from 2001–2002 at the Queen Elizabeth II Health Sciences Centre, Halifax, Nova Scotia, Canada

**Characteristic**	**n (%)**^a^
Sex	
Male	234 (55.7)
Female	186 (44.3)
Age (years)	68.2 ± 13.8
< 65	138 (32.9)
65–79	198 (47.1)
≥ 80	84 (20.0)
History of previous stroke (n = 418)	109 (26.1)
Length of hospital stay (days)	16.2 ± 21.1
Stroke subtype	
Partial anterior circulation	132 (31.4)
Lacunar	106 (25.2)
Posterior circulation	79 (18.8)
Total anterior circulation	30 (7.1)
Transient ischemic attack	59 (14.0)
Uncertain	14 (3.3)
Stroke severity	
Transient ischemic attack	35 (8.4)
Mild	90 (21.5)
Moderate	226 (53.9)
Severe	68 (16.2)
Unknown	1 (0.002)
Disability at discharge	
Independent	333 (79.3)
Dependent	87 (20.7)
Cognitive impairment at discharge	
Not impaired	111 (26.4)
Impaired	84 (20.0)
Missing	225 (53.6)
Atrial fibrillation (n = 418)	60 (14.4)
Average household income (n = 386)	$55,472 ± 19,370
Total medications prescribed^b^	6.4 ± 2.6
Total doses taken per day^b^	8.6 ± 4.5
Monthly cost of prescription drugs^c^	$167.10 ± 99.47

^a^Values expressed as number (%) or mean ± standard deviation, n = 420 unless otherwise noted

^b^Includes all prescription and non-prescription medications ordered by a physician at time of discharge

^c^Includes only community prescription drug costs at time of discharge excluding pharmacist's professional fees

### Medication use and persistence

At discharge, six and 12 months, approximately 25% of the cohort reported the use of an antihyperglycemic (Table 2). Frequency of use in the antihypertensive category ranged from 91% at discharge to 88% at 12 months. Antithrombotic use was above 90% at each time point (or 96%, 93%, and 94%, at discharge, six and 12 months). Fewer patients were prescribed antilipidemic agents (72–75%).

**Table 2:** Medication Use and Persistence by the 420 Patients Enrolled in the Stroke Outcome Study from 2001–2002 at the Queen Elizabeth II Health Sciences Centre, Halifax, Nova Scotia, Canada

**Drug category**	**Users at discharge, n (%)**	**Users at 6 months, n (%)**	**Persistence^a ^at 6 months (%)**	**Users at 12 months, n (%)**^b^	**Persistence^c ^at 12 months (%)**^b^
Antihyperglycemic	104 (24.8)	104 (24.8)	101/104 (97.1)	103 (25.6)	98/101 (97.0)
Insulin	25 (6.0)	26 (6.2)		32 (8.0)	
Oral agents	87 (20.7)	86 (20.5)		82 (20.4)	
Antihypertensive	384 (91.4)	374 (89.0)	363/384 (94.5)	355 (88.3)	348/367 (94.8)
Diuretic	137 (32.6)	140 (33.3)		151 (37.6)	
β-Blocker	182 (43.3)	176 (41.9)		166 (41.3)	
CCB	96 (22.9)	102 (24.3)		96 (23.9)	
ACE-I	317 (75.5)	291 (69.3)		272 (67.7)	
ARB	21 (5.0)	34 (8.1)		40 (10.0)	
Other	7 (1.7)	9 (2.1)		10 (2.5)	
Antithrombotic	405 (96.4)	392 (93.3)	385/405 (95.1)	379 (94.3)	372/387 (96.1)
Antiplatelet^d^	341 (81.7)	333 (79.3)	322/341 (94.4)	322 (80.1)	310/325 (95.4)
Anticoagulant^d^	72 (17.1)	71 (16.9)	60/72 (83.3)	69 (17.2)	57/69 (82.6)
Antilipidemic	307 (73.1)	304 (72.4)	285/307 (92.8)	300 (74.6)	276/302 (91.4)
Statin	306 (72.9)	295 (70.2)		293 (72.9)	
Other	1 (0.2)	10 (2.4)		9 (2.2)	
Combination use					
Antihypertensive and antithrombotic	373 (88.8)	360 (85.7)		342 (85.1)	
Antihypertensive, antithrombotic and antilipidemic	286 (68.1)	284 (67.6)		274 (68.2)	

ACE-I = angiotensin-converting enzyme inhibitor; ARB = angiotensin receptor blocker, CCB = calcium channel blocker

^a^The percentage of patients persistent at six months is the number of patients still receiving a drug from the drug category at six months divided by the total number who were prescribed a drug from the drug category at discharge

^b^At 12 months, 17 patients had died and 1 patient had a missing medication list leaving a total of 402 eligible for analysis

^c^The percentage of patients persistent at 12 months is the number of patients still receiving a drug from the drug category at 12 months divided by the total number who were prescribed a drug from the drug category at discharge **and **who completed the 12 month visit with a medication list

^d^8 patients were prescribed both an anticoagulant and an antiplatelet at discharge

Medication persistence was calculated for the patients who were prescribed the drug at discharge, and was high in all four drug categories at the six and 12 month time points (Table 2). Persistence was highest for the antihyperglycemic drug category (97%) and lowest for the antilipidemics (91%) at one year. Within the antithrombotic category, anticoagulant persistence was lower (83%) compared to antiplatelets (94–95%) at each time point.

### Multivariable analysis

Antihypertensive, antithrombotic and antilipidemic persistence were examined separately (Table 3). Antihyperglycemic agents were not analyzed due to the low frequency of use. Factors significantly associated with antihypertensive nonpersistence were older age, fewer number of medications prescribed at discharge, and lower costs of medications at discharge. Nonpersistence was 1.6% in patients 65–79 years old, and 13.8% in patients 80 years and older (p = 0.0003). Patients with monthly drug costs less than $90.00 per month had a 9.0% nonpersistence rate, while patients with drug costs $200.00 and greater had a 1.4% nonpersistence rate (p = 0.048). In the multivariable model, age and monthly drug costs were significant. Patients 65–79 years old had significantly lower odds of nonpersistence compared to those 80 years and older (adjusted odds ratio (AOR) 0.11, 95% CI 0.03–0.39). In addition, patients were significantly more likely to be nonpersistent with monthly drug costs of < $90 or $90–199.99, compared to those with costs greater than $200 (AOR 6.74, 95% CI 1.32–34.46 for < $90; AOR 5.25, 95% CI 1.14–24.25 for $90–199.99).

**Table 3:** Characteristics Associated with Medication Nonpersistence at Six Months in the 420 Patients Enrolled in the Stroke Outcome Study from 2001–2002 at the Queen Elizabeth II Health Sciences Centre, Halifax, Nova Scotia, Canada

**Characteristic**	**Antihypertensive**			**Antithrombotic**			**Antilipidemic**		
	
	**Not Persistent**^a^	**Unadj OR**	**Adj OR**	**Not Persistent**^a^	**Unadj OR**	**Adj OR**	**Not Persistent**^a^	**Unadj OR**	**Adj OR**
Stroke Cohort	21/384 (5.5)			20/405 (4.9)			22/307 (7.2)		
Age (years)									
< 65	7/112 (6.3)	0.42 (0.16, 1.13)	0.37 (0.13, 1.03)	9/130 (6.9)	0.82 (0.29, 2.29)	0.92 (0.32, 2.65)	Not significant		
65–79	3/192 (1.6)	0.10 (0.03, 0.37)	0.11 (0.03, 0.39)	4/191 (2.1)	0.24 (0.07, 0.83)	0.23 (0.06, 0.81)			
≥ 80	11/80 (13.8)	1.00	1.00	7/84 (8.3)	1.00	1.00			
Total medications prescribed^b^		0.80 (0.65, 0.995)		Not significant			Not significant		
Monthly drug costs^c^									
< $90.00	7/78 (9.0)	7.05 (1.43, 34.81)	6.74 (1.32, 34.46)	Not significant			Not significant		
$90.00 – $199.99	12/161 (7.5)	5.76 (1.27, 26.18)	5.25 (1.14, 24.25)						
≥ $200.00	2/145 (1.4)	1.00	1.00						
OHS impairment before stroke									
Independent	Not significant			17/390 (4.4)	1.00	1.00	Not significant		
Dependent				3/15 (20.0)	5.49 (1.41, 21.27)	7.01 (1.66, 29.58)			
OHS impairment at discharge									
Independent	Not significant			11/318 (3.5)	1.00		Not significant		
Dependent				9/87 (10.3)	3.22 (1.29, 8.04)				
Tobacco use before stroke									
Yes	Not significant			Not significant			5/23 (21.7)	4.35 (1.44, 13.13)	4.35 (1.44, 13.13)
No							17/283 (6.0)	1.00	1.00
History of previous stroke									
Yes	Not significant			Not significant			1/76 (1.3)	0.13 (0.02, 1.004)	
No							21/230 (9.1)	1.00	

Unadj OR = Unadjusted odds ratio (95% confidence interval); Adj OR = adjusted odds ratio (95% confidence interval); OHS = Oxford Handicap Scale

^a^Defined as not continuing to take a medication in the same drug category from discharge to six months, values expressed as number (%)

^b^Includes all prescription and non-prescription medications ordered by a physician at time of discharge

^c^Includes only community prescription drug costs at time of discharge excluding pharmacist's professional fees

Factors associated with antithrombotic nonpersistence were older age, preadmission disability, and disability at discharge (as measured by OHS score) (Table 3). Nonpersistence was 2.1% in patients 65–79 compared to 8.3% in patients 80 years and older (p = 0.029). Patients dependent on others before admission were also more likely to be nonpersistent (20.0%) than those who were independent (4.4%)(p = 0.014). Similarly, patients dependent at discharge were more likely to be nonpersistent than patients who were independent (10.3% vs 3.5%, respectively) (p = 0.012). Age and preadmission disability were significant in the multivariable model. Patients between the ages of 65 and 79 years old were significantly less likely to be nonpersistent than those aged 80 and older (AOR 0.23, 95% CI 0.06–0.81). As well, patients who were disabled before admission had 7.01 higher odds of nonpersistence (95% CI 1.66–29.58) than those who were independent.

Characteristics associated with antilipidemic nonpersistence were negative history of previous stroke, and tobacco use before admission (Table 3). Patients with a history of previous stroke had a nonpersistence rate of 1.3% compared to 9.1% in those without a history (p = 0.05). Patients who reported the use of tobacco before their hospital admission had a higher nonpersistence rate (21.7%) compared to those who did not (6.0%) (p = 0.009). In the multivariable model only tobacco use was found to be significantly associated with antilipidemic nonpersistence (OR 4.35, 95% CI 1.44–13.13, compared to no tobacco use).

## Discussion

The use of pharmacotherapy for secondary stroke prevention at hospital discharge was extremely common in this cohort; 96% of patients were prescribed an antithrombotic and 91% of patients were prescribed at least one antihypertensive. The use of antilipidemic therapy was somewhat less frequent, with 73% prescribed this category at discharge. Medication persistence was exceptionally high with all drug categories exceeding 90% at six months and 12 months. Persistence was highest for antihyperglycemic drugs (97%) and lowest for antilipidemic drugs (91%) at one year. Our findings are consistent with Hamann *et al *who found that 96% of their stroke patients were still receiving an antithrombotic medication at one year.[18] Persistence in the antithrombotic category in their study was 62% for clopidogrel, 84% for ASA, and 77% for warfarin.[18] Our population had better persistence within this class, with 88% of patients still taking an antiplatelet agent and 84% an anticoagulant at one year after stroke. Likewise, Sappok and colleagues found high medication persistence at one year after stroke for antithrombotics (88%), antihypertensives (91%), and antilipidemic therapy (70%).[19]

Medication persistence may be more successful when pharmacotherapy is initiated during the patient's hospitalization, as it was in our cohort. Ovbiagele *et al *found that patients enrolled in the Stroke PROTECT program in a California hospital demonstrated high adherence rates three months after discharge to antithrombotics, statins, and certain antihypertensives.[20] In addition, Aronow *et al *found that starting lipid-lowering therapy in the hospital after patients underwent coronary angioplasty led to better long-term adherence in the EPILOG trial.[37] In the 175 patients who started antilipidemic drugs pre-discharge, 77% were persistent at 6 months, compared to 25% of the 1951 patients who started therapy after discharge.[37]

Persistence may also have been positively affected by the model of care provided by the ASU.[22] Organized stroke unit care has been found to decrease mortality, and increase the likelihood of patients functioning independently and living at home at one year.[38] Follow-up by the stroke team may also have had unmeasured benefits for medication persistence. One-third of patients returned for follow-up visits at the stroke clinic that were unrelated to the research study, although persistence rates were not significantly different among those who returned and those who did not.

We examined by multivariable analysis the factors associated with medication persistence. In our study, patients who did not persist in taking their antihypertensive medications at six months were of older age (80 years and older), were prescribed fewer medications at discharge, and had lower drug costs at discharge. Nonpersistence with antithrombotics was also associated with patients 80 years and older. Older age has not consistently been associated with poor persistence, rather it could be that older persons have more chronic comorbidities and polypharmacy, which puts them at risk of stopping their medications early.[15]

An interesting result was that antihypertensive persistence was better in patients taking more medications. This may be related to the fact that patients often need more than one drug to control their blood pressure, so motivated patients will be on more medications, and in turn their persistence will be higher. In a study of heart failure patients, their adherence to angiotensin-converting enzyme (ACE) inhibitors and antilipidemic drugs improved with increasing number of prescription medications.[39] The authors suggested this could be due to the characteristics of the population such as being highly motivated by the severity of the illness, compliance with follow-up visits, and patient beliefs about the need for medications. As well, increasing medication complexity may require greater attention to medication-taking.[39] In a cohort of new statin users enrolled in a US health care plan, patients were more likely to adhere to their statin therapy when prescribed more concurrent medications.[40]

Another aspect of polypharmacy is medication affordability. We found that higher monthly medication costs were associated with greater persistence, so affordability does not appear to have been a barrier to medication use. One explanation for this may be that in our cohort, most patients over the age of 65 would have drug insurance coverage under the Nova Scotia Senior's Pharmacare Program.[41] Seniors pay an annual premium then a copayment for each prescription. Once the maximum copayment (deductible) is reached, patients do not pay for additional prescriptions.[41]

Disability at admission and at discharge was significantly related to nonpersistence with antithrombotic medications, similar to a finding by Hillen *et al *where non-treatment with antihypertensives or antithrombotics three months after stroke was significantly associated with disability.[17] Sappok *et al*, however, found disability was not related to antithrombotic medication compliance in the multivariable analysis of stroke patients.[19]

An unexpected finding was that antilipidemic nonpersistence was three times higher in patients who used tobacco before their admission compared to those who did not. Sappok *et al *also found that patients with a history of cigarette smoking were less persistent with their antithrombotic treatment at one year after stroke in the univariate analysis, but not after adjusting for other factors.[19] Our results could indicate that tobacco use is associated with socioeconomic indicators that also affect medication-taking. The Heart and Stroke Foundation of Canada found higher rates of tobacco use among Canadians in the low and medium-low income levels.[42] In a report on the use of smoking cessation medications after hospitalization for heart disease, Whelan *et al *found that there were significant socioeconomic differences between patients who did report the use of these medications, and those who were still smokers.[43] Patients who still smoked had less private drug insurance, had more difficulty paying for basic needs, and were less nikely to have finished their secondary school education.[43] In our sample, it was unknown how many patients quit smoking after the stroke. It is also unclear why there is an association between those who smoked and their antilipidemic medication persistence and not with persistence in the other drug classes studied. Further research could help identify the barriers to medication persistence in people who smoke.

There are limitations to the interpretation of our results. Reasons for non-persistence were not formally determined or documented on the medication lists. We have no data on whether patients stopped taking their medications because of out of pocket medication costs; although we attempted to control for this by estimating total monthly drug costs. Some medications may have been appropriately discontinued due to contraindications adverse effects drug interactions lack of efficacy or no longer needed. The elderly may be more susceptible to the toxic effects of drugs and were on many medications which increases the risk of drug interactions. Despite this the importance of these therapies to reduce further stroke morbidity and mortality generally means the majority of patients should be receiving at least one type of medication from a particular category. Clinical data and lifestyle changes were not available in this study so the possibility that some patients reduced their stroke risk and no longer needed medication can not be ruled out.

We were limited to measuring persistence rather than the quality of medication taking according to the prescribed schedule. However persistence is a common useful measurement reported in the literature when other drug use information is not available. Medication use was self-reported and was not verified with another source such as pharmacy administrative claims data. Measuring drug use can utilize direct methods (eg observation or serum drug concentrations) or indirect methods (eg self-report pharmacy refill records)[44]; however a gold standard still does not exist[15]. Patient self-report is a common approach used in clinical settings[44] and has been found to have good concordance (80% 85% and 91%) with pharmacy claims data in studies of antidepressant and cardiovascular medication use [45-47]. Misclassification error in drug use could have occurred if patients were inaccurate with their self-reported medication lists despite attempts by the study coordinator to be as thorough as possible when collecting the data at six and 12 months. Finally the number of patients who were nonpersistent in each drug category was small and variable which resulted in wide confidence intervals and a less precise estimate of effect in the multivariable analysis.

## Conclusion

Self-reported medication persistence rates were high among this cohort of stroke patients enrolled in a prospective study at the Acute Stroke Unit of the QEII HSC in Halifax, Nova Scotia. Physicians, pharmacists and other healthcare providers caring for patients after stroke should recognize factors potentially associated with nonpersistence such as older age fewer prescribed medications disability before and after stroke and previous tobacco use in order to counsel and support patients at risk. The relationships between medication persistence tobacco use and drug costs need further clarification. Improving medication persistence may improve outcomes after stroke and reduce the burden of disease on the healthcare system.

## Competing interests

The authors declare that they have no competing interests.

## Authors' contributions

HLL designed the study, coordinated the data collection, performed the statistical analysis, interpreted the results and drafted the manuscript. ISS, GJG and MRJ assisted with study, design, interpretation of the results and drafting the manuscript. GJF assisted with the statistical analysis, intrepretation of the results and drafting the manuscript. All authors read and approved the final manuscript.

## Pre-publication history

The pre-publication history for this paper can be accessed here:


